# The Value of Serum Tumor Markers and Blood Inflammation Markers in Differentiating Pancreatic Serous Cystic Neoplasms and Pancreatic Mucinous Cystic Neoplasms

**DOI:** 10.3389/fonc.2022.831355

**Published:** 2022-02-25

**Authors:** Huan Wang, Sihai Chen, Xu Shu, Zhijian Liu, Pi Liu, Yong Zhu, Yin Zhu, Huifang Xiong

**Affiliations:** The First Affiliated Hospital of Nanchang University, Nanchang, China

**Keywords:** pancreatic cystic neoplasms, serous cystic neoplasms, mucinous cystic neoplasms, diagnose, CA199

## Abstract

Although many studies have emphasized the prognostic and diagnostic value of tumor markers and various inflammation-related markers, their clinical significance in differentiating benign and malignant pancreatic cystic neoplasms (PCNs) remains to be clarified. The present study explored the value of serum tumor markers and inflammation-related biomarkers in the differentiation of pancreatic serous cystic neoplasms (SCNs) and pancreatic mucinous cystic neoplasms (MCNs). A total of 79 patients with PCNs were included in this study, including 35 patients with SCNs and 44 patients with MCNs. Comparison of baseline data with preoperative results of serum tumor markers and associated inflammatory markers revealed significant differences in carbohydrate antigen 199 (CA199) and “lymphocyte × ALB” (LA) between the two groups (p = 0.0023, p = 0.0149, respectively). Univariate and multivariate regression analyses showed that an increase in CA199 and a decrease in LA were relevant risk factors for MCNs. Finally, the receiver operating characteristic (ROC) curve was generated, and the area under the ROC curve (AUC) was calculated to evaluate the prediction efficiency of each indicator. The results showed that CA199 and LA had good differential diagnostic efficacy for SCNs and MCNs. This is the first to report to demonstrate that LA can be used for the differential diagnosis of SNCs and MCNs.

## Introduction

Pancreatic cystic neoplasms (PCNs) are a group of tumors characterized by cystic lesions formed by pancreatic duct epithelial cells or acinar hyperplasia and retention of pancreatic secretions (1). The latest “European guidelines” suggest that PCN detection rates in the general population range from 2% to 45% (2), and the incidence rate of patients aged 70 years or above reaches 10% (3). PCNs mainly include three clinical subtypes as follows: intraductal papillary mucinous neoplasms (IPMNs), mucinous cystic neoplasms (MCNs), and serous cystic neoplasms (SCNs). Among them, SCNs has no malignant potential and rarely requires surgical treatment. MCNs may deteriorate and are potential malignant tumors that can show a series of biological behavior processes of different degrees of dysplasia and finally to invasive cancer (1). Moreover, due to the differences in biological behavior and the degree of benignity and malignancy between different subtypes, overtreatment or untimely diagnosis and treatment can easily occur, which makes the diagnosis and treatment of PCNs a difficult problem in the clinic. Therefore, there is an urgent need for an accurate preoperative assessment of the benign and malignant degree of PCNs to facilitate subsequent rational clinical treatment. Pathological diagnosis is the gold standard for identifying the nature of PCNs, but it is an invasive operation that causes unnecessary damage to the patient, resulting in certain limitations in clinical application (4).

Numerous studies have shown that laboratory examination is becoming increasingly important to help diagnose PCNs and to distinguish between benign and malignant PCNs. Inflammation plays a key role in the occurrence and progression of tumors. The systemic inflammatory response acts on the occurrence and development of malignant tumors by releasing cytokines and other inflammatory mediators (5–7). Some inflammatory markers based on circulating blood cells can be used as a simple and convenient way to measure the systemic inflammatory response and as independent predictors of survival in a variety of malignancies, including pancreatic cancer (8–10). Recently, there has been much evidence that inflammatory indices, such as the neutrophil-to-lymphocyte ratio (NLR) and platelet-to-lymphocyte ratio (PLR), also play an important role in predicting benign and malignant PCNs (11–13). Tumor markers can reflect the ability of tumor proliferation and metastasis to a certain extent. Studies in recent years have shown that cyst fluid carcinoembryonic antigen (CEA) and cyst fluid carbohydrate antigen 199 (CA199) in PCNs provide great accuracy in distinguishing mucinous and non-mucinous PCNs (14), while the identification value of CEA and CA199 in blood needs to be further studied. The purpose of the present study was to investigate the application value of CEA, CA199, and various inflammatory indicators in the blood in the differential diagnosis of SCNs and MCNs.

## Materials and Methods

### Patients and Methods

In total, 79 patients with PCNs diagnosed at the First Affiliated Hospital of Nanchang University from April 2011 to April 2021 were selected. All patients were diagnosed by surgical pathology or endoscopic ultrasound-guided fine-needle aspiration (EUS-FNA) cytopathology. The results showed that among the 79 patients, 35 had SCNs, and 44 had MCNs. Details of patients are shown in **Supplementary Table 2**. The inclusion criteria were as follows: 1) meet the diagnostic criteria of PCNs; 2) all patients were diagnosed for the first time, and tumor markers and related tests were performed; and 3) gender and age were not limited. The exclusion criteria were as follows: 1) complicated with other pancreatic diseases; 2) combined with other malignant tumors; 3) secondary metastatic carcinoma of pancreas; 4) the nature of the tumor was not confirmed by pathology; and 5) no tumor markers or related tests were performed.

### Data Collection

General information (sex and age), symptoms, preoperative laboratory examination data (CEA, CA199, and inflammatory index in the blood), pathological information, and auxiliary examination information (estimated tumor size and location) were collected.

### Statistical Analysis

Statistical analysis was performed using SPSS 26.0 statistical software (version 1.2.5001) and R language software (R packages “ggplot2”, “pROC”, “Hmisc”, “PerformanceAnalytics”, “corrplot”, “GGally”, and “rms”). For continuous measurement data, such as age, the mean ± standard deviation was used if they were in line with a normal distribution, and a t test was used for comparisons between two groups. Percentiles were used for enumeration data, such as gender, and the chi-square test or Fisher’s exact probability method was used for comparisons between two groups. The receiver operating characteristic (ROC) curve was used to further analyze the diagnostic efficacy of laboratory indices that were meaningful for predicting SCNs and MCNs (15). p < 0.05 was considered statistically significant.

## Results

### Comparison of Preoperative Indices Between SCN and MCN Patients

As shown in **Table 1**, the following 79 patients were enrolled in this study: 35 patients (11 males and 24 females) were confirmed to have SCNs by pathology with an average age of 54.49 ± 12.71 (range, 28–78) years, and 44 patients (11 males and 33 females) were diagnosed with MCNs with an average age of 55.80 ± 15.50 (range, 24–79) years. There was no significant difference in age (p = 0.6878) or sex (p = 0.5266) between the SCN and MCN groups. The average tumor size of the SCN group was 5.44 cm, and the average tumor size of the MCN group was 5.74 cm. There was no significant difference in tumor size between the two groups (p = 0.6725). Additionally, 11/35 SCNs were detected in a head/neck location, and 24/35 SCNs were detected in a body/tail location. Notably, only 8/44 MCNs were detected in a head/neck location, and 36/44 MCNs were detected in a body/tail location. In addition, 21/35 SNC and 32/44 MCN patients had obvious symptoms (such as abdominal pain, abdominal distension, weight loss, fatigue). Among the 44 MCN patients in this study, 8 were pathologically reported to have malignant transformation, and the malignant transformation rate was 18.18%. The preoperative examination of the two groups revealed significant differences in serum CA199 (p = 0.0023) and “lymphocyte × ALB” (LA) (p = 0.0149), but there were no significant differences in other indicators.

**Table 1:** Comparison of preoperative indices between patients with SCNs and MCNs.

Characteristics	SCNs (n = 35)	MCNs (n = 44)	p value
Gender			0.5266
Male	11 (31.43%)	11 (25%)	
Female	24 (68.57%)	33 (75%)	
Age (year) (mean ± SD)	54.49 ± 12.71	55.80 ± 15.50	0.6878
Size (cm) (mean ± SD)	5.44 ± 2.98	5.74 ± 3.32	0.6725
Location			0.1712
Head/neck	11 (31.43%)	8 (18.18%)	
Body/tail	24 (68.57%)	36 (81.82%)	
Complain			0.2317
Symptomatic	21 (60%)	32 (72.73%)	
Asymptomatic	14 (40%)	12 (27.27%)	
CEA (ng/mL)	2.91 ± 4.31	18.00 ± 72.79	0.1771
CA199 (U/mL)	41.73 ± 167.13	236.20 ± 360.23	0.0023*
WBC (×10^9^/L)	6.58 ± 3.29	6.61 ± 3.57	0.9708
Platelet (×10^9^/L)	215.54 ± 64.61	216.86 ± 85.47	0.9398
Lymphocyte (×10^9^/L)	1.61 ± 0.51	1.38 ± 0.57	0.0560
Neutrophil (×10^9^/L)	4.32 ± 3.27	4.65 ± 3.38	0.6603
Monocyte (×10^9^/L)	0.49 ± 0.28	0.46 ± 0.27	0.6317
ALB (g/L)	41.22 ± 4.15	39.47 ± 4.21	0.0717
PLR	145.11 ± 58.28	171.73 ± 71.80	0.0796
NLR	3.60 ± 4.81	4.09 ± 4.24	0.6279
PAR	5.34 ± 1.51	5.60 ± 2.57	0.5966
NAR	0.11 ± 0.10	0.13 ± 0.12	0.5678
LMR	4.10 ± 1.86	3.78 ± 2.00	0.4746
LA	67.49 ± 24.83	54.17 ± 21.98	0.0149*
NP	903.92 ± 607.28	984.57 ± 679.03	0.5844

PLR, platelet–lymphocyte ratio; NLR, neutrophil–lymphocyte ratio; PAR, platelet–ALB ratio; NAR, neutrophil–ALB ratio; LMR, lymphocyte–monocyte ratio; LA, lymphocyte × ALB; NP, neutrophil × platelet. *Means statistically significant.

### Univariate and Multivariate Analyses of Risk Factors Associated With MCNs

According to pathology, the 79 patients were divided into the SCN group and MCN group. Univariate analysis showed that two factors were significantly correlated with MCNs, including increased serum CA199 levels (OR = 1.0034, p = 0.0256) and decreased LA (OR = 0.9752, p = 0.0205). In addition, there was no statistically significant difference (p > 0.05) between SCNs and MCNs in terms of serum CEA level, white blood cell count (WBC), platelets, lymphocytes, neutrophils, monocytes, albumin (ALB), platelet–lymphocyte ratio (PLR), neutrophil–lymphocyte ratio (NLR), platelet–ALB ratio (PAR), neutrophil–ALB ratio (NAR), lymphocyte–monocyte ratio (LMR), or “neutrophil × platelet” (NP) (**Table 2**). The meaningful indexes of univariate analysis were included in logistic regression for multivariate analysis. The results showed that the increase in serum CA199 levels (OR = 1.0031, p = 0.0489) and the decrease in LA (OR = 0.9788, p = 0.0489) were independent predictors of MCNs (**Table 3**). In addition, we analyzed the correlations between all variables and the results were added in the supplementary material (**Supplementary Figure 1**), suggesting that CA199 was related to ALB and CEA. In order to further verify the test efficiency of CA199 and avoid multicollinearity, we selected variables with meaningless correlation to further analyze CA199, and the relevant results were added to the supplementary materials (**Supplementary Table 1**). The results showed that CA199 was still meaningful after calibration of other variables.

**Table 2:** Univariate analysis of risk factors associated with MCNs.

Characteristics	OR	95% CI	p value
Gender	1.3750	0.4892–3.8650	0.5273
Age	1.0065	0.2637–3.8424	0.6835
Size	1.0320	0.2786–3.8228	0.6681
Location	2.0625	1.4626–2.9085	0.1754
Complain	1.7778	1.1240–2.8118	0.2339
CEA	1.0552	0.6726–1.6554	0.2298
CA199	1.0034	0.9544–1.0549	0.0256*
WBC	1.0025	0.1497–6.7148	0.9703
Platelet	1.0002	0.1588–6.2986	0.9388
Lymphocyte	0.4260	0.3739–0.4854	0.0666
Neutrophil	1.0318	0.2850–3.7349	0.6563
Monocyte	0.6718	0.1963–2.2984	0.6276
ALB	0.8998	0.7741–1.0460	0.0768
PLR	1.0065	0.8517–1.1894	0.0852
NLR	1.0261	0.3017–3.4903	0.6246
PAR	1.0608	0.3319–3.3912	0.5929
NAR	3.6114	1.1932–10.9300	0.565
LMR	0.9179	0.3656–2.3045	0.4697
LA	0.9752	0.9367–1.0152	0.0205*
NP	1.0002	0.3211–3.1159	0.5797

PLR, platelet–lymphocyte ratio; NLR, neutrophil–lymphocyte ratio; PAR, platelet–ALB ratio; NAR, neutrophil–ALB ratio; LMR, lymphocyte–monocyte ratio; LA, lymphocyte × ALB; NP, neutrophil × platelet. *Means statistically significant.

**Table 3:** Multivariate analysis of risk factors associated with MCNs.

Characteristics	OR	95% CI	p value
CA199	1.0031	1.0000–1.0062	0.0489*
LA	0.9788	0.9581–0.9999	0.0489*

LA, lymphocyte × ALB. *Means statistically significant.

### Diagnostic Efficacy of the Preoperative Indices in Differentiating SCNs and MCNs

**Figure 1:**
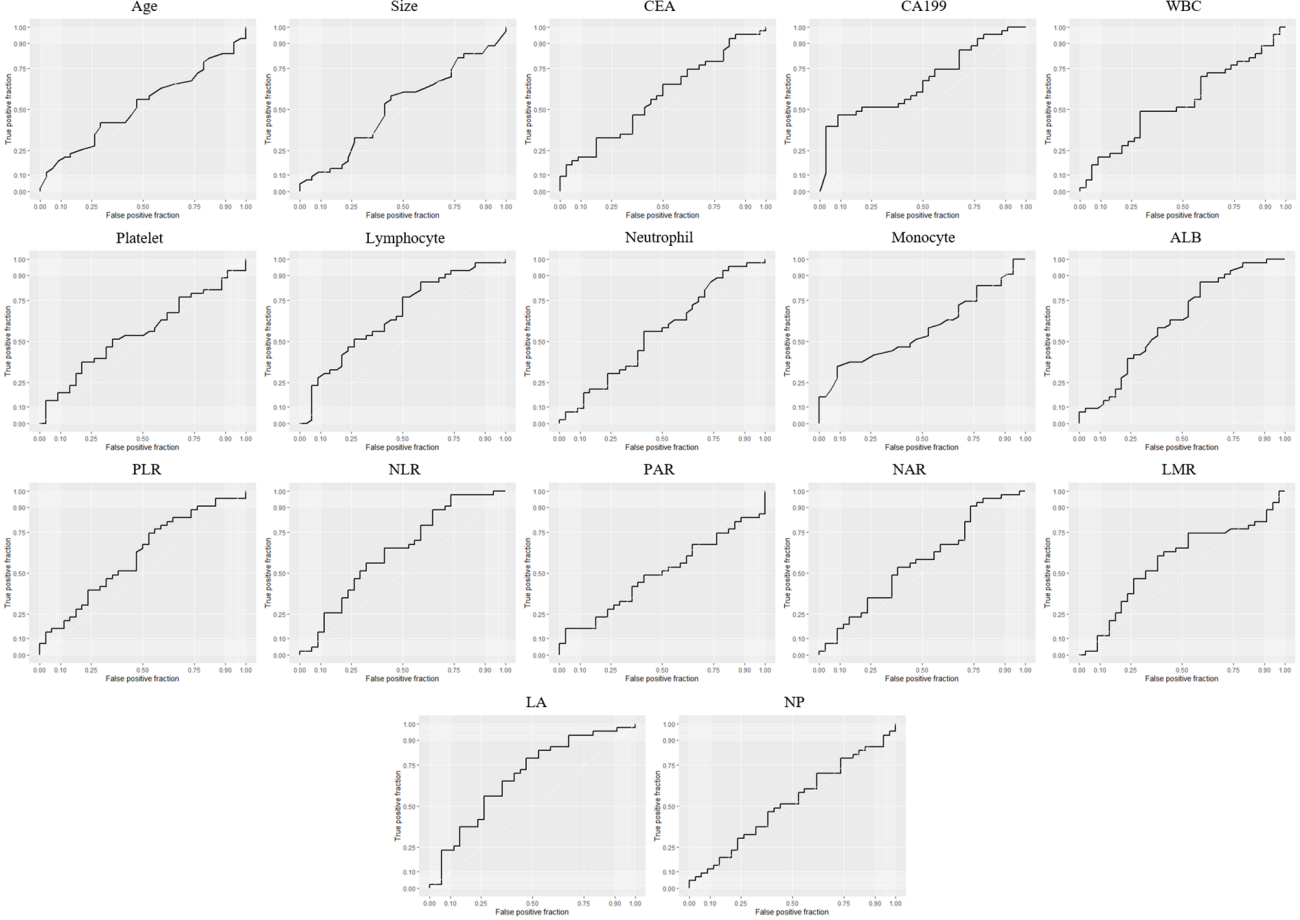
ROC curve of the preoperative indices for differential diagnosis of SCNs and MCNs. PLR, platelet–lymphocyte ratio; NLR, neutrophil–lymphocyte ratio; PAR, platelet–ALB ratio; NAR, neutrophil–ALB ratio; LMR, lymphocyte–monocyte ratio; LA, lymphocyte × ALB; NP, neutrophil × platelet.

ROC curves were generated, and the area under the ROC curve (AUC) was calculated to evaluate the prediction efficiency of each indicator. The ROC curve was drawn with the preoperative indices as test variables and SCNs/MCNs as state variables **(** **Figure 1** **)**. The results of the ROC curve showed that the AUC values of serum CA199 and LA in differentiating SCNs and MCNs were 0.6734 and 0.6765, respectively, indicating that they have a certain value in the differential diagnosis of SCNs and MCNs **(** **Table 4** **)**. According to the Youden index, the diagnostic cutoff point of serum CA199 was 31.315 U/ml (sensitivity, 0.4773; and specificity, 0.9143). The diagnostic cutoff point of LA was 65.45, and the sensitivity and specificity of the differential diagnosis of SCNs and MCNs with LA < 65.45 were 0.7907 and 0.5294, respectively.

**Table 4:** Diagnostic efficacy of the indices in differentiating SCNs and MCNs.

	AUC	95% CI	Cutoff	Sensitivity	Specificity	p value
Age	0.5386	0.4104–0.6668	62.50 year	0.4318	0.7143	0.8662
Size	0.5187	0.3873–0.6501	4.65 cm	0.5909	0.5588	0.6799
CEA	0.5831	0.4561–0.7101	1.745 ng/ml	0.6591	0.5143	0.2399
CA199	0.6734	0.5541–0.7926	31.315 U/ml	0.4773	0.9143	0.0336*
WBC	0.5312	0.3398–0.5979	5.595 × 10^9^/l	0.5000	0.5714	0.9665
Platelet	0.5331	0.4038–0.6624	184.50 × 10^9^/l	0.3636	0.7714	0.8791
Lymphocyte	0.6584	0.5355–0.7813	1.62 × 10^9^/l	0.7727	0.5143	0.0735
Neutrophil	0.5591	0.4288–0.6894	3.38 × 10^9^/l	0.5682	0.6000	0.7078
Monocyte	0.562	0.4342–0.6898	0.30 × 10^9^/l	0.3409	0.9143	0.5695
ALB	0.6228	0.4927–0.7528	43.3000 g/l	0.8605	0.4118	0.0768
PLR	0.6146	0.4878–0.7414	121.02	0.7500	0.4857	0.1177
NLR	0.6403	0.5141–0.7664	2.10	0.6591	0.6000	0.6585
PAR	0.5062	0.3636–0.6241	7.74	0.1628	0.9706	0.5929
NAR	0.567	0.4355–0.6986	0.06	0.9070	0.2647	0.5650
LMR	0.5782	0.4477–0.7088	3.816	0.6136	0.6286	0.5594
LA	0.6765	0.5529–0.8000	65.45	0.7907	0.5294	0.0205*
NP	0.5331	0.4036–0.6626	795.90	0.4773	0.6286	0.6889

PLR, Platelet–lymphocyte ratio; NLR, Neutrophil–lymphocyte ratio; PAR, Platelet–ALB ratio; NAR, Neutrophil–ALB ratio; LMR, Lymphocyte–monocyte ratio; LA, Lymphocyte×ALB; NP, Neutrophil×Platelet. *Means statistically significant.

## Discussion

SCNs and MCNs have different biological characteristics. SCNs are usually benign with only 1% to 3% malignant potential (16), and they can be followed up (17), while MCNs have malignant potential, and surgical resection is recommended after adequate diagnosis (18, 19). Therefore, correctly distinguishing SCNs from MCNs is of great significance for appropriate treatment. Although there are many better methods for preoperative identification of SCNs and MCNs, such as contrast-enhanced endoscopic ultrasonography (20), endoscopic ultrasound-guided fine-needle aspiration of pancreatic cysts (21), and lesion punctures with fluid aspiration followed by through-the-needle biopsies (22), these methods are either invasive or expensive. Therefore, there is an urgent need to find non-invasive, convenient, and inexpensive tests for differential diagnosis.

In this study, the baseline data of the included patients indicated that there were more female patients with SCNs and MCNs than male patients, and the lesions were mostly located in the body or tail of the pancreas, which is consistent with previous research reports (23). At present, many studies have shown that cyst fluid CEA has a good diagnostic effect in differentiating SCNs and MCNs, while blood CEA has a poor diagnostic effect (24). Similarly, the results of the present study also showed that there was no significant difference in serum CEA levels between the SCN group and the MCN group. In contrast, serum CA199 showed good discrimination efficiency. In the present study, the ROC curve was generated, which demonstrated that when serum CA199 was higher than 31.315 U/ml, the occurrence of MCNs was indicated with a sensitivity and specificity of 0.4773 and 0.9143, respectively. The serum tumor marker, CA199 (a tumor-related carbohydrate protein), plays an important role in the diagnosis, treatment, and postoperative follow-up of pancreatic cancer. Increasing evidence indicates that serum CA199 levels play an important role in differentiating the benign and malignant properties of PCNs (25–27). Postlewait et al. (28) analyzed the preoperative blood CA199 level of 349 cases of MCNs and found that the median CA199 level in the malignant group was 210 U/ml; in the nonmalignant group, the median CA199 level was only 15 U/ml (p = 0.001), suggesting that the elevated level of serum CA199 indicated malignant MCNs, which was consistent with the results of the present study.

Recent studies have shown that not only the internal characteristics of tumor cells but also the host inflammatory response determine the occurrence and development of tumors (29). In patients with malignant tumors, host factors, such as weight loss, malnutrition, and systemic inflammatory response, are interrelated, and systemic inflammatory response can be used as a predictor of benign and malignant tumors (30). Some inflammatory indicators (NLR, PLR, and LMR) based on circulating blood cells can be used as a simple and convenient way to measure systemic inflammatory response and as an independent predictor of survival and prognosis in various malignant tumors, including pancreatic cancer (8–10). Recently, there has been much evidence that inflammatory indicators also play an important role in predicting benign and malignant PCNs (12, 13, 31). Our study discovered that LA had good discrimination efficiency between SCNs and MCNs with an AUC of 0.6765 and a diagnostic cutoff point of 65.45. Thus, LA values <65.45 indicate MCNs. Although the potential causal effects behind the association between LA and differential SCNs and MCNs are unclear, the following hypotheses can be proposed. The high density of tumor-infiltrating lymphocytes is closely related to the good prognosis of several cancers, indicating that the antitumor immune response is mainly mediated by lymphocytes (32). Serum ALB is produced by the liver and is known as one of the negative acute phase proteins in response to inflammation. In addition, low ALB concentrations also indicate malnutrition, which can negatively affect tumor immunity in the microenvironment. Given these findings, LA may reflect both the immune response, as represented by lymphocyte count, and nutritional status, as represented by serum albumin levels.

When the two risk factors mentioned above are present at the same time, it indicates a higher risk of MCNs. Surgical treatment and regional pancreatectomy according to imaging results can be considered. In addition, intraoperative frozen pathological results should be considered to prevent more extensive pancreatic parenchymal invasion. Therefore, the present study provides clinicians with a simple, effective, and non-invasive method to identify SCNs and MCNs, thereby facilitating the management and treatment of PCN patients.

The present study had several limitations. First, this study was a retrospective analysis. Only cases with PCNs clearly indicated by surgical pathology or EUS-FNA cytopathology were included, which may have led to selection bias. In addition, due to the limited sample size, we failed to analyze the differential value of these indicators in other types of PCNs, and the predictive efficacy of CA199 and LA still needs to be verified in future clinical work. Importantly, there are an increasing number of studies on PCNs that are exploring the risk factors for the preoperative prediction of malignant PCNs. For clinicians, comprehensive analysis of various risk factors before surgery and accurate balance between the risk of surgery and the risk of malignancy will bring maximum benefits to patients with PCNs.

## Conclusion

In conclusion, as a non-invasive method, tumor markers and inflammatory indicators can complement each other, and joint detection can play an important role in distinguishing SCNs and MCNs. The present study found that serum CA199 and LA can be used independently in the differential diagnosis of SCNs and MCNs. It is worth noting that this is the first report that reveals the value of LA in identifying SCNs and MCNs. The new marker is easily evaluated by routine blood tests, which could provide an opportunity for further investigation.

## Data Availability Statement

The raw data supporting the conclusions of this article will be made available by the authors, without undue reservation.

## Ethics Statement

Ethical approval/written informed consent was not required for the study of animals/human participants in accordance with the local legislation and institutional requirements.

## Author Contributions

HW and SC performed this study and wrote the manuscript as co-first authors; XS and ZL checked the statistics; PL and YoZ revised the manuscript; HX and YoZ designed this study and edited the manuscript. All authors contributed to the article and approved the submitted version.

## Funding

This study was supported by the National Natural Science Foundation of China (No. 82060108) and the Youth Project of the Jiangxi Natural Science Foundation (20202BABL216007).

## Conflict of Interest

The authors declare that the research was conducted in the absence of any commercial or financial relationships that could be construed as a potential conflict of interest.

## Publisher’s Note

All claims expressed in this article are solely those of the authors and do not necessarily represent those of their affiliated organizations, or those of the publisher, the editors and the reviewers. Any product that may be evaluated in this article, or claim that may be made by its manufacturer, is not guaranteed or endorsed by the publisher.
