# Supplementary figures and images for: The Value of Serum Tumor Markers and Blood Inflammation Markers in Differentiating Pancreatic Serous Cystic Neoplasms and Pancreatic Mucinous Cystic Neoplasms
Source: Front Oncol. 2022 Feb 25;12:831355. doi: 10.3389/fonc.2022.831355 (PMC8913928; doi:10.3389/fonc.2022.831355)

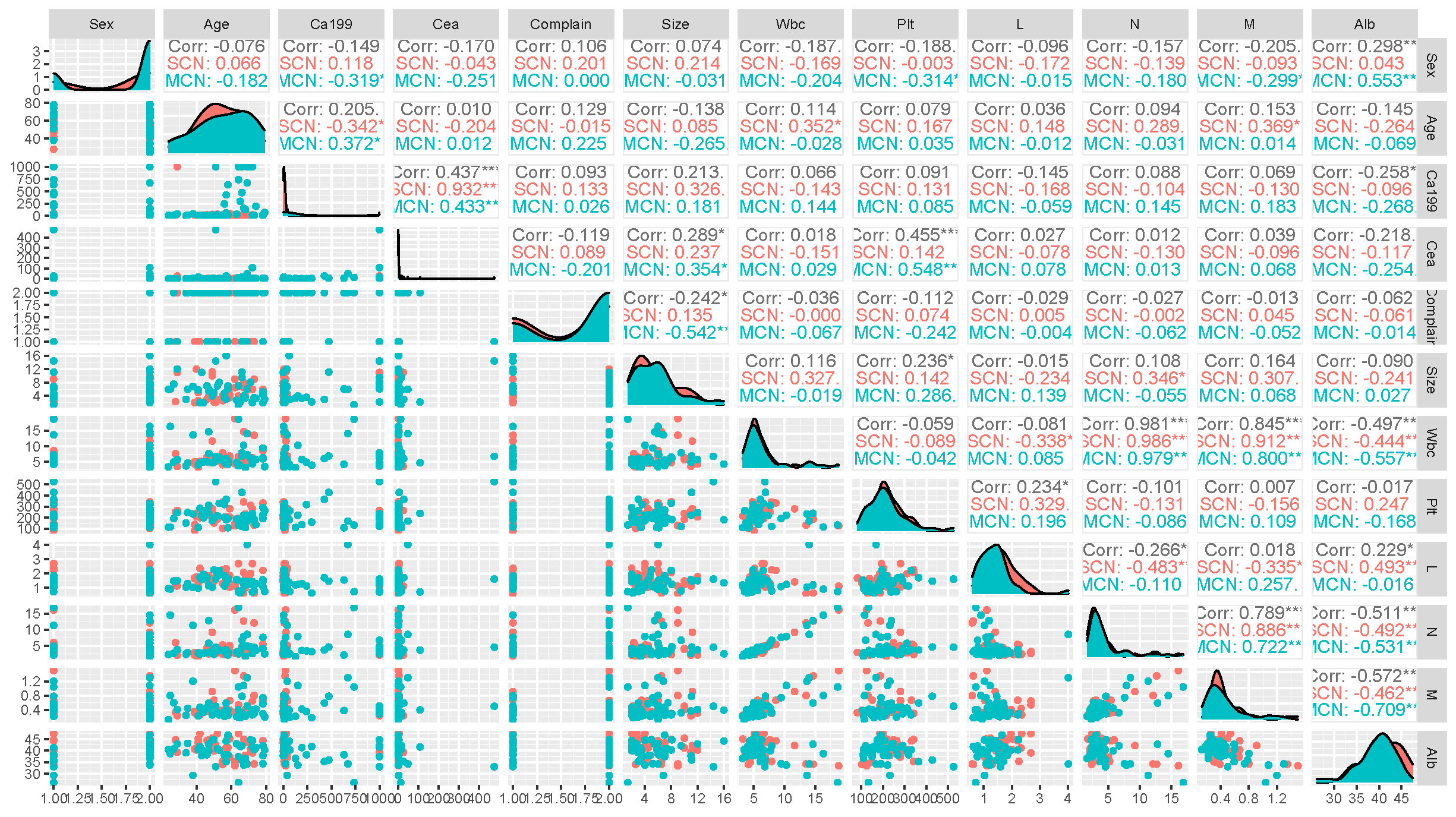

Supplement: Supplementary file 1 [file Image_1.tiff]
