# Supplementary material for: The Value of Serum Tumor Markers and Blood Inflammation Markers in Differentiating Pancreatic Serous Cystic Neoplasms and Pancreatic Mucinous Cystic Neoplasms
Source: Front Oncol. 2022 Feb 25;12:831355. doi: 10.3389/fonc.2022.831355 (PMC8913928; doi:10.3389/fonc.2022.831355)
Supplement: Supplementary file 2 [file Table_1.docx]

| Variable | Adjust OR | Adjust P value |
| --- | --- | --- |
| Gender |  |  |
| CA199 | 1.0039 (1.0006,1.0072) | 0.0217 |
| Age |  |  |
| CA199 | 1.0035 (1.0004,1.0065) | 0.02631 |
| CEA |  |  |
| CA199 | 1.0032 (1,1.0065) | 0.05187 |
| Complain |  |  |
| CA199 | 1.0033 (1.0004,1.0062) | 0.02806 |
| Size |  |  |
| CA199 | 1.0034 (1.0004,1.0063) | 0.02673 |
| WBC |  |  |
| CA199 | 1.0034 (1.0004,1.0064) | 0.02509 |
| Platelet |  |  |
| CA199 | 1.0034 (1.0004,1.0064) | 0.02564 |
| Lymphocyte |  |  |
| CA199 | 1.0036 (1.0004,1.0068) | 0.02921 |
| Neutrophil |  |  |
| CA199 | 1.0034 (1.0004,1.0064) | 0.02656 |
| Monocyte |  |  |
| CA199 | 1.0036 (1.0005,1.0067) | 0.0237 |
| ALB |  |  |
| CA199 | 1.0029 (0.9999,1.0058) | 0.0541 |

Supplementary Table 1 The test efficiency of CA199 after adjusting for each variable
